# Effect of Laparoscopic Total Extraperitoneal Umbilical Hernia Repair on Incision Infection, Complication Rate, and Recurrence Rate in Patients with Umbilical Hernia

**DOI:** 10.1155/2022/7055045

**Published:** 2022-01-11

**Authors:** Zhao Zhang, Li Li, Bo Liu, Fengen Wang, Wenli Wang, Xian Liu, Yanmei Ju

**Affiliations:** Surgical Gucheng County Hospital of Hebei Province, Hengshui 053000, China

## Abstract

The aim of this study is to clarify the influence of laparoscopic total extraperitoneal umbilical hernia repair on incision infection, complication rate, and recurrence rate in patients with an umbilical hernia (UH). Sixty-seven UH patients referred to our hospital from June 2017 to June 2019 were selected as the research participants. Thirty-six patients in the research group (RG) were treated with laparoscopic total extraperitoneal umbilical hernia repair, and the other 31 cases in the control group (CG) were treated with traditional umbilical hernia repair. The two cohorts of patients were compared with respect to the curative effect after treatment; intraoperative blood loss, operation time, postoperative pain time, ambulation time, and hospital stay; incidence of complications; pain severity (VAS) before and after operation; sleep quality (PSQI) before and after operation; patient satisfaction after treatment; and recurrence half a year after discharge. The RG presented a higher effective treatment rate (*P* < 0.05), less intraoperative blood loss, operation time, postoperative pain time, ambulation time, and hospital stay, as well as lower incidence of complications than the CG (*P* < 0.05). VAS and PSQI scores differed insignificantly between the two cohorts of patients before treatment (*P* > 0.05) but reduced after treatment, with lower VAS and PSQI scores in the RG than in the CG (*P* < 0.05). The number of people who were highly satisfied, as investigated by the satisfaction survey, was higher in the RG than in the CG, while the recurrence rate of prognosis was lower than that in the CG (*P* < 0.05). Laparoscopic total extraperitoneal umbilical hernia repair is effective for UH patients and can validly reduce the incidence of complications and recurrence rate, which has huge clinical application value.

## 1. Introduction

Umbilical hernia (UH) refers to an abdominal external hernia in which the contents of the abdominal cavity protrude outward through the weak area of the umbilical region, which is mainly characterized by soft protuberance or protrusion of the umbilicus and is more common in infants while less prevalent in adults [1]. Adult umbilical hernia (AUH) is defined as the extra-abdominal hernia occurring in the umbilical region of adults, which usually occurs in adults aged 35–50, with the ratio of female to male of about 3 : 1 [2]. The pathogenesis of AUH is due to the increase of intra-abdominal pressure and the gradual expansion of abdominal organs after the closure of the umbilical ring, resulting in the formation of hernia [3]. The main presentation is umbilical mass with or without local pain [4]. AUH cannot heal by itself, and surgical treatment is the only and most effective method for a cure [5]. Some patients with intestinal incarceration without timely operation may develop serious diseases such as intestinal perforation and necrosis, and even death, which is of high risk [6]. Hence, surgical treatment as soon as possible is of utmost importance for patients. As to the treatment, traditional umbilical hernia repair is often used in early clinical treatment, but it predisposes patients to complications and poor prognosis [7]. Therefore, the clinic has been trying to find a more effective treatment.

With the continuous development of medical technology, minimally invasive surgery has gradually replaced traditional open surgery in the medical field [8]. Laparoscopic surgery has been gradually selected and recognized by more patients because of its advantages of aesthetic wound, less injury, fast postoperative recovery, short hospital stay, light postoperative pain, and low postoperative recurrence rate [9]. Looking up the previous data, it is found that laparoscopic total umbilical hernia repair has gradually increased in recent years. Evidence has shown that it has low complications, causes no scrotal edema, and rarely induces urinary retention [10]. Other research suggests that it is especially suitable for the treatment of inguinal hernia, recurrent hernia, and incisional hernia with weak transverse fascia, with favourable treatment effects [11]. However, there are few clinical reports on laparoscopic total extraperitoneal umbilical hernia repair for UH patients. Accordingly, this paper analyzes the influence of laparoscopic total extraperitoneal umbilical hernia repair on incision infection, complication rate, and recurrence rate in UH patients, so as to provide an effective reference for future clinical treatment of AUH.

## 2. Materials and Methods

### 2.1. General Information

Sixty-seven patients with UH admitted to our hospital from June 2017 to June 2019 were selected as the research participants, among which 36 patients were treated with laparoscopic total extraperitoneal umbilical hernia repair as the research group (RG), and 31 patients were treated with traditional umbilical hernia repair as the control group (CG). The baseline data such as age and BMI were similar in the two cohorts (*P* > 0.05; Table 1). The internal ethics committee approved this study protocol, and all the enrolled participants have signed the informed consent.

### 2.2. Inclusion and Exclusion Criteria

The inclusion criteria were as follows: patients with first diagnosis and confirmed diagnosis of UH by preoperative imaging and physical examination, aged 30 to 60 years, with complete data. The exclusion criteria were as follows: patients with chronic cough, patients with constipation, patients with other serious diseases, patients with severe infectious diseases, patients with contraindications to surgery or drugs, and patients with mental disorders or poor treatment compliance.

### 2.3. Methods

RG: After general anesthesia with endotracheal intubation, the patient's lower extremities were separated by about 30°, the appropriate patch was selected, and the urinary catheter was indwelled before the operation. The gap between the rectus abdominis and the posterior rectus abdominis was established by reverse puncture, and the pneumoperitoneum was established by puncturing using the Veress needle in the abdomen. Then, the pneumoperitoneum was routinely filled with 12 mm Hg carbon dioxide pneumoperitoneum to control the pressure at 1.6 kPa. Thereafter, the laparoscope was placed into the abdominal cavity and observed. According to the observation, another 2-3 laparoscopes were placed, and an ultrasonic scalpel or electrocoagulation hook was sent for separation to further enlarge the preperitoneal space. Medical instruments such as ultrasonic scalpel and grasping forceps were used to separate the great omentum and the intestinal canal attached to the hernia sac, and care was taken to avoid cutting the peritoneum. After the hernia ring was exposed, the separation range was properly adjusted according to the required mesh size. The mesh was first rolled into a roll and then paved in the abdominal cavity, and the inner surface of the hernia sac was treated by electrocoagulation. Then, the incision was sutured with the previously reserved suture, the knot position was placed in the subcutaneous tissue, and the mesh was suspended on the abdominal wall. After that, cotton pads were placed locally for compression bandaging to eliminate the hernia sac mesh gap and relieve the pneumoperitoneum. Finally, the incision was sutured and bandaged with an abdominal belt.

CG: The preoperative preparation was the same as that of the RG. The subcutaneous tissue and hernia sac were incised, and the distance between the posterior sheath and peritoneum was measured. After that, a mesh of the same area was cut to fill, and the surrounding tissue was sutured. This is followed by drainage tube indwelling after flushing with normal saline. Finally, the incision was sutured layer by layer.

### 2.4. Endpoints

The curative effect after treatment; intraoperative blood loss, operation time, postoperative pain time, ambulation time and hospital stay; incidence of complications; pain (visual analogue scale, VAS) before and after operation; sleep quality (Pittsburgh Sleep Quality Index, PSQI) before and after operation; patient satisfaction after treatment; and recurrence of patients 6 months after discharge.

### 2.5. Statistical Methods

All the statistical analyzes of the experimental results were carried out by using SPSS25.0, and all the graphical results were visualized by using GraphPad8. Data were given (mean ± standard deviation); the *t*-test was employed for intergroup comparisons, one-way analysis of variance (ANOVA) and LSD post-test were applied for multigroup comparisons, and repeated measures ANOVA and Bonferroni posthoc test were adopted for multiple time points. A *P* value less than 0.05 was considered to be significant.

## 3. Results

### 3.1. Comparison of Baseline Data

Comparison of age, gender, BMI, hernia ring diameter, living environment, exercise habits, smoking history, drinking history, diabetes history, and ethnicity between the two cohorts showed no statistically significant differences (*P* > 0.05).

### 3.2. Comparison of Post-Treatment Curative Effects

In the RG, 18 patients were cured, 16 were effective, and 2 were ineffective, and the effective treatment rate was 94.44%. In the CG, 10 patients were cured, 14 were effective, and 7 were ineffective, with an effective treatment rate of 83.87%. The effective treatment rate in RG was statistically higher than that in CG (*P* = 0.042), Table 2.

### 3.3. Comparison of Clinical Indexes

**Figure 1:**
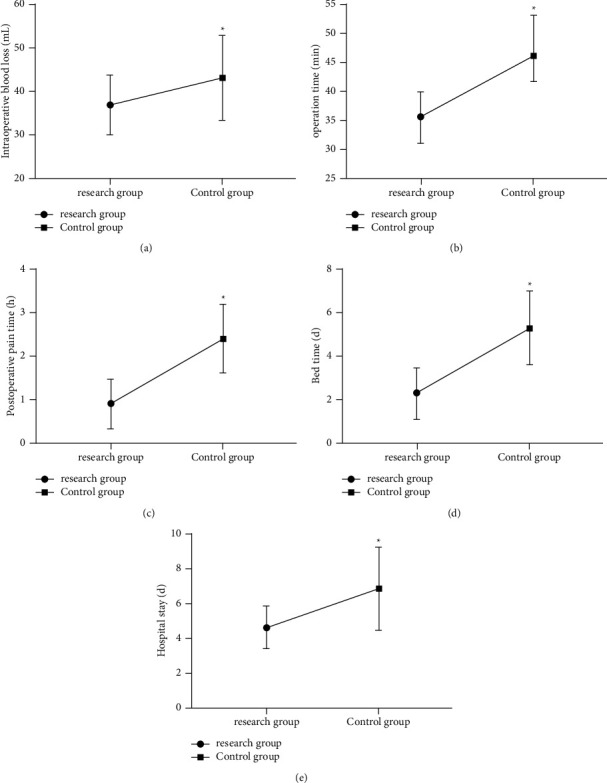
Comparison of clinical indexes. (a) Comparison of intraoperative blood loss between the two groups. (b) Comparison of operation time between the two groups. (c) Comparison of postoperative pain time between the two groups. (d) Comparison of ambulation time between the two groups. (e) Comparison of hospital stay between the two groups. *Note.*^*∗*^*P* < 0.05.

The statistical analysis of the operation indexes revealed evidently less intraoperative blood loss, operation time, postoperative pain time, ambulation time, and hospital stay in the RG than the CG (*P* < 0.05).  Figure 1.

### 3.4. Incidence of Complications in the Two Groups

The incidence of post-treatment complications such as incision infection, hematoma, ileus, thrombosis, and pneumonia in the two groups was observed. The results identified a distinctly lower incidence of complications in the RG (8.33%) than the CG (29.03%) (*P* = 0.028).  Table 3.

### 3.5. Pain Score (VAS) and Sleep Quality (PSQI) Assessments in the Two Groups

**Figure 2:**
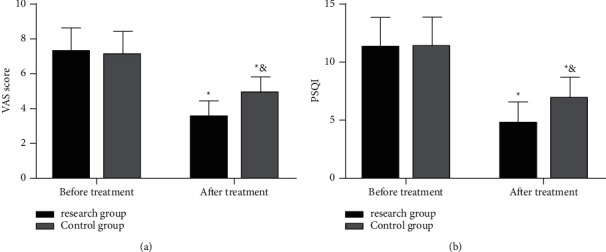
Pain score (VAS) and sleep quality (PSQI) assessments in the two groups. (a) VAS scores in the two groups. (b) PSQI scores in the two groups. *Note.*^*∗*^*P* < 0.05 vs. before treatment and *P* < 0.05 vs. the research group.

VAS and PSQI were employed to evaluate the pain and sleep quality of the two cohorts of patients before and after treatment. Statistical differences were absent with respect to VAS and PSQI scores between the two cohorts before treatment but were present after treatment, with more evident reductions in both scores in the RG than in the CG (*P* < 0.05).  Figure 2.

### 3.6. Patient Satisfaction after Treatment

Comparing patient satisfaction after treatment, it was found that there was no distinct difference in the number of patients who rated satisfied, improvement needed, and dissatisfied between the RG and CG (*P* > 0.05), while the number of patients who were highly satisfied was evidently higher in the RG (*P* = 0.001).  Table 4.

### 3.7. Recurrence in Patients in the Two Groups after Discharge

The patients were followed up for half a year after discharge. The results showed no recurrence in the RG while 5 in the CG within 6 months. The recurrence rate in the RG was significantly lower than that in the CG, with a statistical difference (*P* = 0.012).  Table 5.

## 4. Discussion

The main symptoms of AUH are indigestion, abdominal discomfort, etc., and then, there will be pain in the abdomen, which is usually dull pain [12]. Most people who suffer from UH need to be treated by surgery [12]. There are many triggers for AUH, but the main reasons are excessive abdominal wall traction and increased intra-abdominal pressure [13]. After giving birth, most women will become obese with too much fat in the body, and sometimes develop symptoms such as a cough, which may lead to increased intra-abdominal pressure [14]. When coughing, the internal pressure rises, the internal organs of the body will be squeezed, and the small intestine and other organs will penetrate the abdominal wall, which is likely to cause the disease [14]. Attributed to the advantages of less trauma, quick postoperative recovery and low recurrence rate, the laparoscopic technique is increasingly widely used. Therefore, this paper studies the influence of laparoscopic total extraperitoneal umbilical hernia repair on incision infection, complication rate, and recurrence rate in UH patients.

We first compared the baseline data of enrolled UH patients before the trial was initiated. The data revealed no statistical difference between the two cohorts of patients regarding age, sex, BMI, hernia ring diameter, living environment, exercise habit, smoking history, drinking history, diabetes history, and ethnicity, suggesting the feasibility of follow-up experiments. Comparison of the curative effect between the two groups of patients after treatment demonstrated that the effective treatment rate was 94.44% in the RG (18 cured, 16 effective, and 2 ineffective treatments), which was markedly higher than that in the CG (83.87%; 10 cured, 14 effective, and 7 ineffective treatments). Through placing the mesh in the preperitoneal space and applying the reverse puncture technique to place the cannula, laparoscopic total extraperitoneal umbilical hernia repair will not damage the important blood vessels and has no serious impact on the abdominal wall function, which may be one of the prime reasons for the superior efficacy in the RG over the CG [15]. Author Edelman showed that laparoscopic total extraperitoneal umbilical hernia repair is markedly effective in treating AUH [16], which supports our findings. Then, the clinical indicators were compared and determined: less intraoperative blood loss, operation time, postoperative pain time, ambulation time, and hospital stay in the RG than in the CG. It suggests that the application of laparoscopic total extraperitoneal umbilical hernia repair can effectively improve the therapeutic effect of AUH, reduce the pain time of patients, promote disease recovery, effectively control the blood loss, and reduce the infection rate with less trauma, which has a positive impact on promoting the rehabilitation of patients. Laparoscopic total extraperitoneal umbilical hernia repair is a method that can be used to simultaneously manage a variety of unilateral and bilateral inguinal hernias without increasing incisions, which effectively avoids aggravating the patient's pain [15]. Moreover, the natural structure of the groin area will not be damaged during operation, which accords with the principle of tension-free repair, so the clinical symptoms of patients can be effectively improved, the operation time can be reduced, and the incidence of adverse reactions can be controlled. This is also consistent with the role of laparoscopic total extraperitoneal umbilical hernia repair mentioned in previous studies [17, 18]. Besides, postoperative complications such as wound infection, hematoma, ileus, thrombosis, and pneumonia were observed in the two groups. The results showed that the total complication rate in the RG was 8.33%, which was notably lower than the 29.03% in the CG, further corroborating the above experiments and reflecting the high efficacy of laparoscopic total extraperitoneal umbilical hernia repair on AUH patients. Previous literature has shown that the placement of the mesh after closing the hernia ring during repair can effectively reduce the recurrence rate and the incidence of complications [19], which is similar to the results of this study. Furthermore, the VAS score for pain evaluation and PSQI score for sleep quality assessment were similar in the RG and the CG before treatment, while both scores in the RG decreased and were notably lower than those in the CG after treatment. We speculate that the reason lies in the fact that laparoscopic total extraperitoneal umbilical hernia repair does not require extensive dissection and separation of abdominal wall tissues for placement of meshes, so the postoperative pain is light, the recovery is quick, and the risk of complications is effectively avoided, contributing to meliorated sleep quality. By investigating patient satisfaction, we found that there were observably more patients in the RG who were highly satisfied with the operation, which further reflects the effectiveness and safety of laparoscopic total extraperitoneal umbilical hernia repair. We also followed up the patients for half a year after discharge, and the results revealed no recurrence in the RG, which proved the application value of laparoscopic total extraperitoneal umbilical hernia repair for AUH patients.

## 5. Conclusion

The purpose of this research is to try to clarify the influence of laparoscopic total extraperitoneal umbilical hernia repair on incision infection, complication rate, and recurrence rate in UH patients. However, there are still some deficiencies to be addressed due to limited experimental conditions. For example, there are many treatment methods available for UH in clinical practice, and there are still great controversies regarding the selection of the best therapeutic methods for the disease; thus, there may be some discrepancies in the experimental results when other methods rather than traditional umbilical hernia repair are used as the control. In addition, this study did not carry out tailored treatment for UH patients with different severity, which warrants further experimental analysis. We will expand the sample size, extend the experimental period, and conduct more detailed and comprehensive experimental analyses to obtain more accurate experimental results.

In conclusion, laparoscopic total extraperitoneal umbilical hernia repair is effective for the treatment of UH and can effectively reduce the incidence of complications and recurrence rate in UH patients, which is of huge clinical application value.

## Figures and Tables

**Table 1:** General data of patients [*n* (%)].

	Research group (*n* = 36)	Control group (*n* = 31)	*t* or *χ*^2^	*P*
Age (years old)			1.115	0.269
	42.7 ± 3.2	43.6 ± 3.4		
Gender			0.061	0.806
Male	15 (41.67)	12 (38.71)		
Female	21 (58.33)	19 (61.29)		
BMI (kg/cm^2^)			0.401	0.690
	24.12 ± 3.23	24.46 ± 3.71		
Hernia ring diameter (cm)			0.189	0.851
	4.64 ± 2.63	4.52 ± 2.55		
Living environment			0.129	0.720
Urban	17 (47.22)	16 (51.61)		
Rural	19 (52.78)	15 (48.39)		
Exercise habits			0.113	0.910
Yes	10 (27.78)	9 (29.03)		
No	26 (72.22)	22 (70.97)		
History of smoking			0.110	0.741
Yes	13 (36.11)	10 (32.26)		
No	23 (63.89)	21 (67.74)		
History of drinking			0.104	0.747
Yes	16 (44.44)	15 (48.39)		
No	20 (55.56)	16 (51.61)		
History of diabetes			0.037	0.848
Yes	4 (11.11)	3 (9.68)		
No	32 (88.89)	28 (90.32)		
Ethnicity			1.413	0.235
Han	35 (97.22)	28 (90.32)		
Ethnic minorities	1 (2.78)	3 (9.68)		

**Table 2:** Comparison of clinical efficacy between the two groups [*n* (%)].

	Research group (*n* = 36)	Control group (*n* = 31)	*χ* ^2^	*P* value
Cured				
	18 (50.00)	10 (32.26)		
Effective				
	16 (44.44)	14 (45.16)		
Ineffective				
	2 (5.56)	7 (22.58)		
Total effective rate			4.152	0.042*∗*
	34 (94.44)	26 (83.87)		

**Table 3:** Comparison of incidence of complications between the two groups [*n* (%)].

	Research group (*n* = 36)	Control group (*n* = 31)	*χ* ^2^	*P* value
Incision infection				
	1 (2.78)	4 (12.90)		
Hematoma				
	1 (2.78)	1 (3.23)		
Ileus				
	0 (0.00)	1 (3.23)		
Thrombosis				
	1 (2.78)	2 (6.45)		
Pneumonia				
	0 (0.00)	1 (3.23)		
Incidence of complications (%)			4.854	0.028
	8.33	29.03		

**Table 4:** Comparison of treatment satisfaction between two groups [*n* (%)].

	Research group (*n* = 36)	Control group (*n* = 31)	*χ* ^2^	*P* value
Highly satisfied			11.490	0.001^*∗*^
	23 (63.89)	7 (22.58)		
Satisfied			3.025	0.082
	10 (27.78)	15 (48.39)		
Improvement needed			1.740	0.187
	3 (8.33)	6 (19.35)		
Dissatisfied			2.394	0.122
	0 (0.00)	2 (6.45)		

**Table 5:** Recurrence in patients in the two groups after discharge [n(%)].

	Research group (*n* = 36)	Control group (*n* = 31)	*χ* ^2^	*P* value
Recurrence rate (%)			6.257	0.012^*∗*^
	0 (0.00)	5 (16.13)		

## Data Availability

The datasets used and/or analyzed during the current study are available from the corresponding author on reasonable request.
